# Heart Failure with Reduced Ejection Fraction and Prognostic Scales: The Impact of Exercise Modality in Cardiopulmonary Exercise Tests

**DOI:** 10.3390/jcm11113122

**Published:** 2022-05-31

**Authors:** Julia Herrero Huertas, Marta García Clemente, Beatriz Díaz Molina, José Luis Lambert Rodríguez, Marta Íscar Urrutia

**Affiliations:** 1 Department of Pneumology, Fundación Jiménez Díaz University Hospital, 28040 Madrid, Spain; herrerohuertas@gmail.com; 2 Department of Pneumology, Asturias Central University Hospital, 33011 Oviedo, Spain; martaiscar@gmail.com; 3 Department of Cardiology, Asturias Central University Hospital, 33011 Oviedo, Spain; beadimo@gmail.com (B.D.M.); joseluis.lambert@gmail.com (J.L.L.R.)

**Keywords:** heart failure with reduced ejection fraction, prognostic scales, VO_2_, VE/VCO_2_ slope, cardiopulmonary exercise tests, treadmill, cycle ergometer

## Abstract

The cardiopulmonary exercise (CPET) test is an essential tool to determine the severity, prognosis, and need for invasive treatments in heart failure with reduced ejection fraction (HFrEF) but disregards the exercise modality. The present study aimed at analyzing the differences between treadmill and cycle-ergometer exercises. This was a prospective study, involving 65 patients with HfrEF who performed treadmill exercise followed by cycle-ergometer exercise 72 h later. We enrolled 65 patients, aged 58 ± 9 years, with an ejection fraction of 29 ± 9%. Peak VO_2_ was 20% greater (95% CI: 18–21%; *p* < 0.000) on the treadmill, and the ventilatory efficiency estimated by the VE/VCO_2_ slope (32 ± 8 vs. 34 ± 9; *p* < 0.05). The ventilatory response was greater on the treadmill: maximum ventilation (55 ± 16 vs. 46 ± 11 L/min; *p* < 0.000) and ventilatory reserve at the maximum effort (28 ± 17 vs. 41 ± 15%; *p* < 0.000). These values led to a change in the functional class of 23 (51%) patients and ventilatory class of 28 (47%) patients. Differences in the main parameters, including peak VO_2_ and VE/VCO_2_, impact prognostic scales and possible advanced treatments; therefore, the results should be interpreted in accordance with the exercise modality.

## 1. Introduction

Exercise intolerance in heart failure with reduced ejection fraction (HFrEF) results from changes in oxygen delivery to skeletal muscles and its utilization by myocytes [1]. Cardiopulmonary exercise tests (CPETs) demonstrate this imbalance between oxygen supply and demand, particularly decreased oxygen consumption (VO_2_) and maximum workload [2].

CPET is an objective, reproducible and non-invasive test [3] for the functional capacity of patients with HFrEF, routinely used in the baseline and follow-up assessments of this disease to determine its severity, prognosis and need for invasive treatments [4]. The gold-standard quantifier of functional capacity in CPET is VO_2_ [5].

The most common exercise modalities in CPET are the treadmill and cycle ergometer, but protocols vary with the objective and limitation and/or weakness of the subject being studied [6,7]. Advantages of the treadmill stress test are familiarity with the exercise modality, participation of a higher number of muscle groups and greater work against gravity, thereby increasing the stress to which the systems involved in response to exercise are subjected [7,8]. For all these factors, peak VO_2_ is 5–16% higher with treadmill than with cycle ergometer tests [9,10,11]. This difference in peak VO_2_ may occur in diseases whose prognostic stratification is based on this parameter, among other factors [2]. Disadvantages of the treadmill are determined by difficulties in the exact quantification of the work to which the patient has been subjected and relationship between the speed–slope and the metabolic cost [7]. The other CPET modality, the cycle ergometer, minimizes artifacts, is more affordable, and requires less space compared to the treadmill, but its main advantage is to facilitate quantification of the external work to which the patient is subjected [7]. Conversely, the cycle ergometer leads to lower-limb fatigue in untrained participants and lower VO_2_ compared to the treadmill, among other disadvantages [12].

The analysis of peak VO_2_ for determining prognostic scale scores [13] and decision making regarding the indication of advanced therapies, such as heart transplantation [14] or left ventricular assist devices (LVADs) [4,15], disregards the exercise modality, assuming that the oxygen requirement is identical in both tests. This assumption can lead to error by underestimating the real functional class of the patient.

Recently, parameters in addition to peak VO_2_ have been incorporated into the prognostic stratification of HFrEF, such as the VE/VCO_2_ slope [16] and exercise oscillatory ventilation [17] (EOV). These parameters have become increasingly relevant for obtaining predictive information, regardless of the patient’s effort.

To date, few studies involving a small sample size of select patients have compared the results from both exercise modalities using the parameters peak VO_2_ and VE/VCO_2_ slope [18,19,20,21,22,23,24].

The aim of the present study was to analyze differences in CPET parameters between exercise modalities (treadmill and cycle ergometer) and their impact on the functional assessment and severity stratification of patients with HFrEF.

## 2. Materials and Methods

We prospectively enrolled outpatients with heart failure from the Asturias Central University Hospital (Hospital Universitario Central de Asturias—HUCA). All of them were in a clinically stable condition, undergoing current optimal treatment, and were referred for CPET from July 2019 to March 2020.

Inclusion criteria were left ventricular ejection fraction (LVEF) < 40% and New York Heart Association functional classes I–III [25]. Exclusion criteria included: (a) hospitalization within the last 3 months for decompensated heart disease or uncontrolled atrial fibrillation, (b) patients suffering any severe event during the CPETs or the cooldown period as myocardial ischemia and/or severe arrhythmia (ventricular tachycardia and ventricular fibrillation), and (c) not having completed both CPETs.

Demographic, anthropometric, comorbidity, and treatment data for all patients included in the study were recorded.

The study protocol was approved by the Research Ethics Committee of the Principality of Asturias (registration number 174/19). All patients included in the study received a patient information sheet and signed the informed consent form for participation in the study.

### 2.1. CPET

Each patient performed two CPETs up to their maximum tolerance. The first CPET was performed on a treadmill (HP Cosmos Pulsar 2002, Nußdorf, Germany), using a modified version of the protocol developed by Bruce [26], comprising 1 min stages with increasing speeds ranging from 2.7 to 6 km/h and progressive incline from 0% to 16%. The second CPET was performed 72 h later on a cycle ergometer (Corival Lode BV, Groninga, Netherlands) with 5–20 W/min increments adjusted to the functional limitation of each patient. Before the second test, the patients were evaluated to ensure the absence of changes in symptoms, physical findings, weight, and medication.

In addition to performing the breath-by-breath gas analysis (Ergocard, Medisoft Exp’Air 1.25, Breeze, Belgium) averaging the data every 10 s, the oxygen saturation and heart rate (HR) were continuously monitored by pulse oximetry and 12-lead electrocardiography, respectively. Every 2 min, blood pressure (BP) was manually recorded, and degrees of dyspnea and lower-limb fatigue were assessed using the modified Borg scale (0–10) [27]. The ergospirometer was calibrated before each test [12].

HR (bpm), oxygen saturation (%), VO_2_ (mL/min), CO_2_ production (VCO_2_; mL/min) and minute ventilation (VE; L/min) were recorded continuously. The following values were calculated: respiratory quotient (RQ), ventilatory equivalents for O_2_ and CO_2_ (EqO_2_ and EqCO_2_), VE/VCO_2_ slope, ventilatory threshold (VT) using the system of ventilatory equivalents [6], breathing reserve (BR = (1 − [VE/maximal voluntary ventilation]) × 100) [6], end-tidal CO_2_ and O_2_ pressures (PetCO_2_ and PetO_2_; mmHg), O_2_ pulse (VO_2_/HR; mL/bpm), and the presence of EOV, which was measured as cyclic oscillations in ventilation during ≥60% of exercise with an amplitude ≥15% of the mean value at rest [6]. Maximum values were recorded as those averaged over the last 20 s of the test. The HR recovery index (HRR-1; bpm) calculated the difference between the maximum HR during exercise and the HR 1 min after completing the test [28].

The criteria used to end the test were the maximum effort perceived by the supervised patient, serious cardiovascular events, and presence of limiting symptoms. In the recovery phase, monitoring was maintained for 3 min [6].

Functional limitation was rated using the Weber classification [29]. This classification divides patients with HFrEF as functions of the peak VO_2_/kg and prognosis: class A, peak VO_2_/kg ≥ 20 min/mL/kg; class B, peak VO_2_/kg = 16–20 min/mL/kg; class C, peak VO_2_/kg = 10–15 min/mL/kg; and class D, peak VO_2_/kg ≤ 10 min/mL/kg. Patients in class A have the best prognosis.

The ventilatory classification was proposed by Arena et al. [30], which divides patients into four classes as functions of the VE/VCO_2_ slope and prognosis: class I, VE/VCO_2_ slope < 30; class II, ≥30 VE/VCO_2_ slope < 36; class III, ≥36 VE/VCO_2_ slope < 45; and class IV, VE/VCO_2_ slope ≥ 45. Patients with ventilatory class I have the best prognosis.

### 2.2. Spirometry

Prior to CPET, spirometry was performed in a seated position to record the forced expiratory volume in the first second (FEV1), the forced vital capacity (FVC), and FEV1/FVC ratio. These parameters were interpreted using the theoretical reference values of the Global Lung Initiative [31]. Obstruction was defined as having a post-bronchodilator FEV1/FVC ratio lower than the lower limit of normal (LLN) [32]. The diagnosis of chronic obstructive pulmonary disease (COPD) was conducted following the criteria of the Global Initiative for Chronic Obstructive Lung Disease (GOLD) [33]. Maximal voluntary ventilation (MVV) was estimated using the formula FEV1 × 40 [6].

### 2.3. Statistical Analysis

Data analysis was conducted using the statistical program Stata (Version 15.4.2, StataCorp, College Station, TX, USA). A descriptive analysis was initially performed. Numerical data are expressed as numbers and percentages. The quantitative variables are expressed as the mean and standard deviation. The paired t-test was used to compare continuous data. Differences between proportions were analyzed using the Pearson chi-squared test. Correlations between peak VO_2_ in each exercise modality were evaluated by calculating the Pearson correlation. A *p*-value of ≤0.05 was considered as statistically significant.

## 3. Results

From the 80 patients initially referred for the study, 15 were excluded; 10 did not fulfill the inclusion criteria and the other 5 did not complete the two CPETs previously described. Finally, we enrolled a total of 65 patients, including 49 (75%) men and 16 (25%) women, with a mean age of 57.8 ± 9.3 years. The mean LVEF was 29.5% ± 8.6%. Table 1 outlines the characteristics of the participants.

Comorbidities included atrial fibrillation with controlled ventricular response in 12 (18%) patients and COPD in 18 (28%) patients. COPD was mild and moderate in 44% and 50% of the patients, respectively.

### 3.1. CPET

The time interval between the two CPETs was 3.0 ± 1.9 days. Table 2 shows the detailed results of both CPETs.

### 3.2. Effort Level

The exercise duration and maximum RQ were similar in both exercise modalities. RQ reached 1.04 ± 0.05 and 1.03 ± 0.06 with the treadmill and cycle ergometer, respectively (*p* = 0.43), without significant differences, suggesting a similar degree of effort.

The treadmill exercise was suspended by four (6%) patients, because of dyspnea in two patients and hypertensive crisis in the other two patients. The cycle ergometer test was suspended by 15 (23%) patients because of lower-limb fatigue in five (33%) patients, hypertensive crisis in three (20%) patients, dyspnea in three (20%) patients, and other reasons in four (27%) patients.

### 3.3. VO_2_

**Figure 1:**
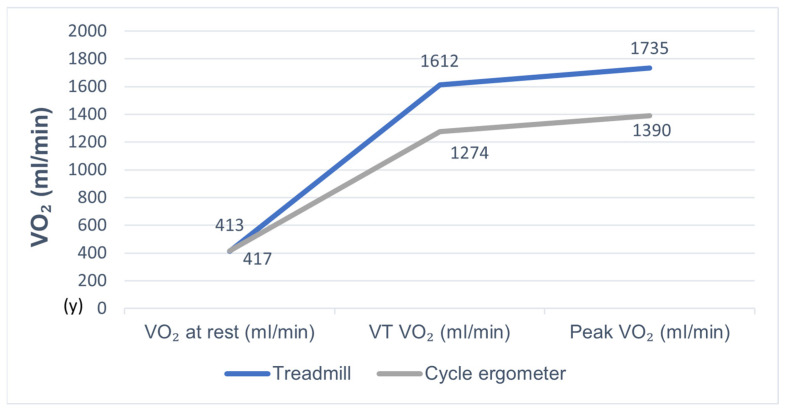
VO_2_ variation during CPET.

Regarding variations in VO_2_ during CPET, baseline VO_2_ (subject at rest) did not differ significantly between the two modalities. However, VT and peak VO_2_ reached during exercise differed significantly between the two modalities (*p* < 0.000); Table 3; Figure 1.

The peak VO_2/_kg in treadmill and cycle ergometer exercises was 21.0 ± 5.1 and 16.9 ± 4.1 mL/min/kg, respectively, showing a significant difference (*p* < 0.000).

The peak VO_2_ and peak VO_2_/kg were 20% (95% confidence interval: 18–21%) higher value in the treadmill exercise than in the cycle ergometer exercise (*p* < 0.000). The correlation between peak VO_2_ and peak VO_2_/kg was excellent (r = 0.93 vs. 0.89, *p* < 0.000) in both ergometers.

### 3.4. Cardiovascular Response

The maximum HR was higher in the treadmill exercise than in the cycle ergometer exercise (117.3 ± 15.7 vs. 107.5 ± 15.2 bpm; *p* < 0.000), with a better HRR-1 (22.4 ± 12.8 vs. 16.4 ± 8.8 bpm; *p* < 0.000) in patients with a sinus rhythm (*n* = 53).

The O_2_ pulse (VO_2_/HR) in VT was 14.6 ± 3.6 and 12.5 ± 3.3 mL/bpm in the treadmill and cycle ergometer exercises, respectively, showing a significant difference (*p* < 0.000).

In the BP response throughout the test, systolic BP did not differ at the maximum exercise capacity, but diastolic BP at the maximum effort was significantly lower in the treadmill exercise than in the cycle ergometer exercise (81.5 ± 16.6 vs. 87.4 ± 16.2 mmHg; *p* < 0.000). In both tests, patients showed a hypertensive response to exercise, with systolic BP > 220 mmHg or diastolic BP > 120 mmHg in four patients (*p* = NS).

**Figure 2:**
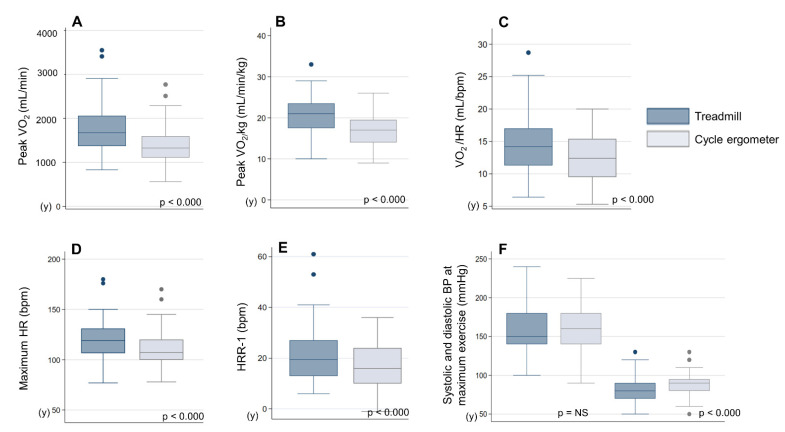
Boxplot of cardiovascular variables and VO_2_ on the treadmill and cycle ergometer. (**A**) Peak VO_2_ (mL/min), (**B**) peak VO_2/_kg (mL/min/kg), (**C**) VO_2_/HR at VT (mL/bpm), (**D**) maximum HR (bpm), (**E**) HRR-1 (bpm), (**F**) systolic and diastolic BP at the maximum exercise (mmHg). VO_2_: oxygen consumption; HR: heart rate; HRR-1: HR recovery index; BP: blood pressure; NS: not significant.

Figure 2 shows a comparison of the results of cardiovascular variables by ergometer.

### 3.5. Ventilatory Response and Gas Exchange

The ventilatory response was greater on the treadmill than on the cycle ergometer, at both the maximum respiratory rate (30.5 ± 5.6 vs. 27.3 ± 5.0 rpm; *p* < 0.000) and maximum VE (54.7 ± 16.3 vs. 45.8 ± 11.4 L/min; *p* < 0.000). Lower EqO_2_ (35.6 ± 4.9 vs. 37.0 ± 5.5; *p* < 0.05) and EqCO_2_ (36.3 ± 5.0 vs. 37.4 ± 5.4; *p* < 0.05), higher ΔPetCO_2_ (3.8 ± 4.0 vs. 2.3 ± 4.7; *p* < 0.05), and lower VE/VCO_2_ slope (32.2 ± 7.9 vs. 33.9 ± 8.8; *p* < 0.05) were also observed, reflecting a better ventilatory efficiency on the treadmill than on the cycle ergometer.

BR at the maximum effort was significantly lower on the treadmill than on the cycle ergometer (28.4 ± 17.1 vs. 41.1 ± 15.2%; *p* < 0.000).

Finally, EOV was present in 19 (30%) patients during the treadmill exercise and in 23 (36%) patients during the cycle ergometer exercise, with no significant differences (*p* = NS).

**Figure 3:**
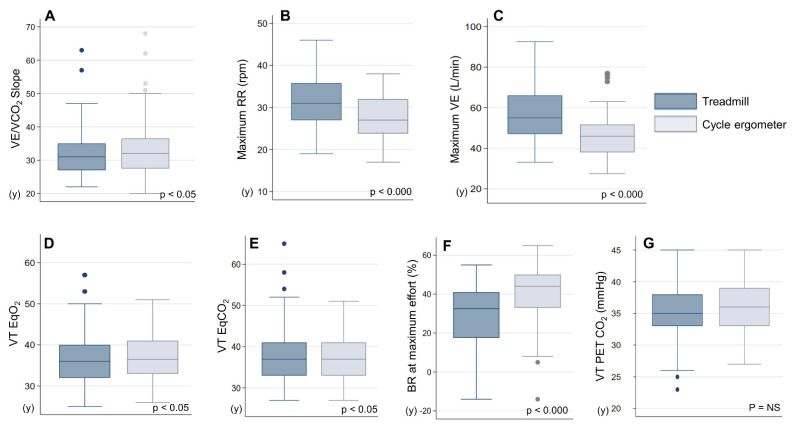
Boxplot of respiratory variables on the treadmill and cycle ergometer. (**A**) VE/VCO_2_ Slope, (**B**) maximum RR (rpm), (**C**) maximum VE (L/min), (**D**) VT Eq O_2_, (**E**) VT Eq CO_2_, (**F**) BR at maximum effort (%), (**G**) VT PET CO_2_ (mmHg). RR: respiratory rate; VE: ventilation; VT: ventilatory threshold; EqO_2:_ oxygen equivalent; EqCO_2_: carbon dioxide equivalent; BR: breathing reserve; P_ET_CO_2_: partial pressure end-tidal carbon dioxide; NS: not significant.

Figure 3 shows a comparison of the results of respiratory variables by ergometer.

### 3.6. Prognostic Classification

**Figure 4:**
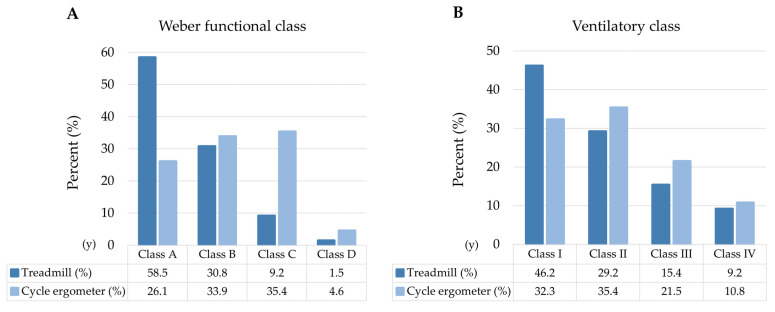
(**A**) Analysis of the Weber functional class as a function of peak VO_2_/kg and ergometer. (**B**) Ventilatory class according to VE/VCO_2_ slope and ergometer.

The Weber functional [29] and ventilatory [30] classifications were used for prognostic purposes. The Weber functional class varied as a function of peak VO_2_/kg. The ergometer showed a change in functional class in 36 (55%) participants, with a descent on the scale during the cycle ergometer exercise (Figure 4A).

The ventilatory class as a function of VE/VCO_2_ slope and ergometer changed in 31 (48%) patients, with 10 (15%) patients displaying a worse ventilatory efficiency on the treadmill and 21 (32%) on the cycle ergometer (Figure 4B).

### 3.7. Symptomatology and Preferences

The symptomatology of the patients according to the Borg questionnaire [27] throughout the test showed higher dyspnea values on the treadmill than on the cycle ergometer (5.4 ± 2.4 vs. 3.7 ± 2.3; *p* < 0.000) and higher lower-limb fatigue values on the cycle ergometer than on the treadmill (3.8 ± 3.1 vs. 4.8 ± 2.4; *p* < 0.05).

Regarding ergometer preference, 32 (50%) patients opted for the cycle ergometer, 26 (41%) opted for the treadmill, and six (9%) were indifferent to the type of ergometer (*p* = NS). The reasons for preferring the cycle ergometer were the greater sense of security and less dyspnea at the end of the test. The reason for preferring the treadmill was the greater familiarity with the type of exercise.

## 4. Discussion

The exercise modality in the CPET may affect the final VO_2_, changing the functional class and, accordingly, the therapeutic approach [13,34]. In our study, the peak VO_2_ and peak VO_2_/kg of patients with HFrEF were significantly higher when performing the CPET on the treadmill than on the cycle ergometer, with a 20% difference in both parameters, despite using protocols with a similar workload, as shown by no significant differences in RQ or test duration between the two ergometers.

The impact of this difference on VO_2_ was determined by prognostic scales [13,34], such as the Weber functional classification [29], as these functional classes changed in more than half of the patients, depending on the ergometer; and by current treatment guidelines for HFrEF because the most extended cutoff peak VO_2_/kg for considering the use of advanced therapies, such as LVAD or heart transplantation [4,14,15], is 12 mL/kg/min, without indicating the exercise modality of the CPET. In our study, peak VO_2_/kg was lower than 12 mL/kg/min in 1 (1.5%) patient on the treadmill and in 11 (17%) patients on the cycle ergometer. The same patient may show different values depending on the ergometer. Therapeutic recommendations are based on these values, irrespective of the ergometer used in the CPET.

The VE/VCO_2_ slope was lower, indicating a higher ventilatory efficiency when performing the test on the treadmill. In addition, the ventilatory class [30] changed in half of the patients as a function of the ergometer used in the CPET.

Regarding the ventilatory response, VE, RR, and BR reflected the higher ventilatory demand of the treadmill, although with a better ventilatory efficiency when analyzing PetCO_2_ and EqO_2_/EqCO_2_. Of these parameters, only PetCO_2_ had been studied in HFrEF by Mazaheri et al. [24], although in a small sample of exclusively male patients (*n* = 30) with significantly lower effort on the treadmill. These results highlight the importance of the exercise modality in ventilatory demand because the ventilatory response varies with the type of ergometer.

EOV may be a prognostic factor of HFrEF [13], in addition to VO_2_ and VE/VCO_2_ slope. However, no studies have assessed whether its presence varies as a function of exercise modality in HFrEF or not. In our study, the prevalence of EOV did not differ significantly, despite a non-significant tendency towards a higher prevalence with the cycle ergometer, which must be verified in future research.

The hemodynamic response in different exercise modalities was described by Kim et al. [20], in a study involving 18 patients with heart failure showing a higher cardiac output and a greater A–V difference in oxygen in treadmill tests as explanatory variables of the differences in VO_2_. In our study, the higher O_2_ pulse, as an indirect measure of the systolic volume and higher HR on the treadmill, corroborated the findings of Kim et al. [20] Using a higher number of muscle groups increases the metabolic requirement during exercise, and, consequently, the cardiac output and peak VO_2_. This phenomenon, together with the increase in catecholamines during the treadmill exercise and changes in blood flow distribution in HFrEF [20], may explain the higher HR on the treadmill and higher diastolic BP on the cycle ergometer. Regarding HRR-1, previous studies [22,23,24] reported disparate results. In our study, HRR-1 was better on the treadmill.

Finally, the patients’ preference for the ergometer did not differ significantly, but they felt more secure on the cycle ergometer and more familiar with the type of exercise on the treadmill.

A few studies compared the two ergometers in the 1990s [18,19,20], with a small sample size of select patients, as in Witte et al. [21] (*n* = 11), Maeder et al. [22] (*n* = 21), Beckers et al. [23] (*n* = 55) and Mazaheri et al. [24] (*n* = 30). VO_2_ differences ranged from 10% to 23% [18,19,20,21,22,23,24] and with considerable variability in VE/VCO_2_ slope [21,22,23,24]. These differences may be related to the characteristics of the selected patients, small sample size, and differences between protocols, in addition to the respiratory behavior and its repercussion on related parameters, such as BR, PetCO_2_, EqO_2_, EqCO_2_, and EOV. The patient’s preference for the exercise modality was also overlooked in those studies.

The strengths of the present study are its broader population sample than those published earlier and analysis of a higher number of parameters than those published in other studies, including ventilatory parameters, such as BR, EqO_2_, EqCO_2_, and the presence of EOV, which had not been previously compared in HFrEF. Similarly, another strength of this study was the level of effort achieved with both exercise modalities, which was similar. This study was limited by its single-center setting. Future multicenter studies should be performed to corroborate the results. The lack of randomization could be considered a limitation, although the symptoms, weight, or medication did not change when performing the CPET according to the usual practice of our center: first on a treadmill and after 72 h on a cycle ergometer. Finally, our sample represented as many patients treated in routine practice as possible and included patients with chronic obstructive pulmonary disease, which could serve as a reference for new studies performing subgroup analyses.

## 5. Conclusions

Treadmill exercise produces a higher peak VO_2_, with a higher ventilatory and cardiovascular response. In diseases whose prognostic classification partly depends on CPET parameters, such as HFrEF, the results of this test should be interpreted considering not only sex, age, disease, and comorbidity but also the ergometer used in the CPET.

## Figures and Tables

**Table 1:** Baseline characteristics of the included patients (*n* = 65).

	Mean (SD)/No (%)
Age (years)	57.8 ± 9.3
Sex (male)	49 (75%)
Body mass index (kg/m^2^)	29.2 ± 4.4
Comorbidity - Atrial fibrillation - COPD Mild Moderate Severe Very severe	12 (18%) 18 (28%) 8 (44%) 9 (50%) 1 (6%) 0 (0%)
Functional class - NYHA class I - NYHA class II - NYHA class III - NYHA class IV	13 (20%) 41 (63%) 11 (17%) 0 (0%)
Cause of HFrEF - Ischemic - Idiopathic - Hereditary - Other	28 (44%) 27 (42%) 6 (9%) 4 (5%)
LVEF (%)	29.5 ± 8.6
Laboratory values - Hemoglobin (g/dL) - Creatinine (mg/dL) - Glomerular filtration rate (mL/min/1.73 m^2^)	14.05 ± 1.78 1.19 ± 0.8 71.6 ± 19.09
Spirometry (mL/% theoretical) - FEV_1_ - FVC - FEV_1_/FVC ratio	2701 ± 757 (84 ± 16%) 3762 ± 1020 (90 ± 16%) 71 ± 7%
Chronic treatment - Beta-blocker - ACE-I - ARB - Mineralocorticoid receptor antagonist - Angiotensin receptor-neprilysin inhibitor - Loop diuretic - Thiazides - Ivabradine - Digoxin - Amiodarone	63 (97%) 37 (57%) 27 (41%) 37 (57%) 22 (34%) 45 (69%) 2 (3%) 7 (11%) 1 (2%) 4 (6%)
Implantable cardioverter defibrillator - Single chamber - Cardiac resynchronization therapy	34 (52%) 11 (32%) 23 (68%)

COPD: chronic obstructive pulmonary disease; NYHA: New York Heart Association; HFrEF: heart failure with reduced left ventricular ejection fraction; LVEF: left ventricular ejection fraction; FEV_1_: maximum expiratory volume in the first second; FVC: forced vital capacity; ACE-I: angiotensin converting enzyme inhibitor; ARB: angiotensin AT_1_-receptor blocker.

**Table 2:** Cardiopulmonary exercise testing parameters.

CPET Variable	Treadmill	Cycle Ergometer	*p* Value
**Test Duration (minutes)**	10.5 ± 2.7	10.2 ± 3.1	NS
**Reached VT (%)**	93.8%	76.9%	*p* < 0.05
**RQ Max**	1.04 ± 0.05	1.03 ± 0.06	NS
**HR at Rest (bpm)**	69.6 ± 10.9	68.8 ± 11.0	NS
**HR at Maximum Effort (bpm)**	117.3 ± 15.7	107.5 ± 15.2	*p* < 0.000
**HR Recovery Index (bpm)**	22.4 ± 12.8	16.4 ± 8.8	*p* < 0.000
**SBP at Rest (mmHg)**	123.3 ± 21.6	125.3 ± 21.8	NS
**SBP at the End of Exercise (mmHg)**	158.7 ± 33.5	161.4 ± 30.9	NS
**DBP at Rest (mmHg)**	75.8 ± 10.8	78.9 ± 12.2	*p* < 0.05
**DBP at the End of Exercise (mmHg)**	81.5 ± 16.6	87.4 ± 16.2	*p* < 0.000
**Initial O_2_ Saturation (%)**	97.0 ± 1.3	97.2 ± 1.2	NS
**Final O_2_ Saturation (%)**	96.4 ± 1.8	97.2 ± 1.4	*p* < 0.000
**Resting VO_2_ (mL/min)**	412.7 ± 162.2	416.6 ± 166.1	NS
**Peak VO_2_ (mL/min)**	1734.9 ± 549.9	1390.5 ± 427.0	*p* < 0.000
**Peak VO_2/_kg (mL/min/kg)**	21.0 ± 5.1	16.9 ± 4.1	*p* < 0.000
**VO_2_ at VT (mL/min)**	1612.5 ± 382.5	1274.0 ± 361.8	*p* < 0.000
**VCO_2_ at VT (mL/min)**	1587.7 ± 376.2	1259.0± 354.3	*p* < 0.000
**O_2_ pulse at VT (mL/bpm)**	14.6 ± 3.6	12.5 ± 3.3	*p* < 0.000
**VE/VCO_2_ Slope**	32.2 ± 7.9	33.9 ± 8.8	*p* < 0.05
**VE Max (L/min)**	54.7 ± 16.3	45.8 ± 11.4	*p* < 0.000
**RR Max (rpm)**	30.5 ± 5.6	27.3 ± 5.0	*p* < 0.000
**EqO_2_ at VT**	35.6 ± 4.9	37.0 ± 5.5	*p* < 0.05
**EQCO_2_ at VT**	36.3 ± 5.0	37.4 ± 5.4	*p* < 0.05
**P_ET_CO_2_ at Rest (mmHg)**	32.0 ± 4.4	32.8 ± 4.9	NS
**P_ET_CO_2_ at VT (mmHg)**	36.2 ± 4.2	36.1 ± 4.5	NS
**ΔP_ET_CO_2_** **(mmHg)**	3.8 ± 4.0	2.3 ± 4.7	*p* < 0.05
**BR at Maximum Effort (%)**	28.4 ± 17.1	41.1 ± 15.2	*p* < 0.000
**Exercise Oscillatory Ventilation** **(%)**	29.6	36.5	NS
**Final Borg Dyspnea (1–10)**	5.4 ± 2.4	3.7 ± 2.3	*p* < 0.000
**Final Borg Lower Limbs (1–10)**	3.8 ± 3.1	4.8 ± 2.4	*p* < 0.05

CPET: Cardiopulmonary exercise test; VT: ventilatory threshold; RQ: respiratory quotient; HR: heart rate; SBP: systolic blood pressure; DBP: diastolic blood pressure; VO_2_: oxygen consumption; VCO_2_: CO_2_ production; VE: ventilation; RR: respiratory rate; EqO_2:_ oxygen equivalent; EqCO_2_: carbon dioxide equivalent_;_ P_ET_CO_2_: partial pressure end-tidal carbon dioxide; ΔP_ET_CO_2_: P_ET_CO_2_ increase from start of test to VT; BR: breathing reserve; NS: not significant.

**Table 3:** VO_2_ variation during CPET.

	Treadmill	Cycle Ergometer	*p*-Value
VO_2_ at rest	412.7 ± 162.2	416.6 ± 166.1	NS
VT VO_2_	1612.5 ± 382.5	1274.0 ± 361.8	<0.000
Peak VO_2_	1734.9 ± 549.9	1390.5 ± 427.0	<0.000

## Data Availability

Not applicable.
